# Chronic Respiratory Disease and Health-Related Quality of Life of African American Older Adults in an Economically Disadvantaged Area of Los Angeles

**DOI:** 10.3390/ijerph16101756

**Published:** 2019-05-17

**Authors:** Mohsen Bazargan, James L. Smith, Paul Robinson, John Uyanne, Ruqayyah Abdulrahoof, Chika Chuku, Shervin Assari

**Affiliations:** 1 Departments of Family Medicine, College of Medicine, Charles R Drew University of Medicine and Science, Los Angeles, CA 90059, USA; mobazarg@cdrewu.edu (M.B.); jamessmith@cdrewu.edu (J.L.S.); paulrobinson@cdrewu.edu (P.R.); 2 Departments of Public Health, College of Health and Science, Charles R Drew University of Medicine and Science, Los Angeles, CA 90059, USA; ruqayyahabdulrahoof@cdrewu.edu (R.A.); chikachuku@cdrewu.edu (C.C.); 3 Departments of Family Medicine, University of California, Los Angeles (UCLA), Los Angeles, CA 90095, USA; 4 Division of Internal Medicine & Geriatrics, Charles R Drew University of Medicine and Science, Los Angeles, CA 90059, USA; johnuyanne@cdrewu.edu

**Keywords:** African Americans, Blacks, older adults, chronic disease, chronic medical conditions, chronic respiratory conditions (CRC), asthma, chronic bronchitis, health-related quality of life

## Abstract

*Background.* Most of the attention of policy makers, program planners, clinicians, and researchers in the area of physical health disparities among African American older adults has been traditionally focused on cardiometabolic disease and cancer. Among a long list of chronic medical conditions, chronic respiratory conditions (CRCs), such as asthma, chronic bronchitis, and emphysema, have received less attention. *Purpose.* This study investigated whether CRCs contribute to physical and mental health-related quality of life (HRQoL) of African American older adults who live in economically disadvantaged urban areas, and whether these effects are due to demographic factors, socioeconomic status (SES), health behaviors, and comorbid medical and mental conditions. *Methods.* This community-based study recruited 617 African American older adults (age ≥ 65 years) from Service Planning Areas (SPA) 6, an economically disadvantaged area in South Los Angeles. Structured face-to-face interviews were used to collect data on demographic factors (age and gender), SES (educational attainment and financial difficulty), living arrangements, marital status, health behaviors (cigarette smoking and alcohol drinking), health (CRC, number of comorbid medical conditions, depressive symptoms, and pain intensity), and physical and mental HRQoL (Physical and Mental Component Summary Scores; PCS and MCS; SF-12). Linear regressions were used to analyze the data. *Results.* The presence of CRCs was associated with lower PCS and MCS in bivariate analysis. The association between CRCs and PCS remained significant above and beyond all confounders. However, the association between CRCs and MCS disappeared after controlling for confounders. *Conclusion.* For African American older adults living in economically disadvantaged urban areas, CRCs contribute to poor physical HRQoL. Evaluation and treatment of CRCs in African American older adults may be a strategy for reduction of disparities in HRQoL in this population. As smoking is the major modifiable risk factor for CRCs, there is a need to increase accessibility of smoking cessation programs in economically disadvantaged urban areas. More research is needed on the types, management, and prognosis of CRCs such as asthma, chronic bronchitis, and emphysema in African American older adults who reside in low-income and resource limited urban areas.

## 1. Introduction

### 1.1. Background

Few studies have focused on the social, behavioral, psychological, and medical determinants of the Health-Related Quality of Life (HRQoL) of African American older adults [1,2], particularly those who are classed as low income and live in economically disadvantaged urban areas [2,3]. Most of what we know about the HRQoL of African American older adults is specific to a particular clinical sample [4,5]. This is mainly because most of the research on the HRQoL of African Americans is conducted in homogenous samples of patients with a particular illness such as breast [4,5] or prostate [6] cancer, or at most individuals with any type of cancer [7], or in cancer survivors [8,9]. Thus, we need more information on determinants of HRQoL in the general population of economically disadvantaged African American older adults [10]. Such research may generate new knowledge that can potentially introduce novel venues to enhance HRQoL of low socioeconomic status (SES) African American older adults who have multimorbidity and live in economically challenged urban areas [2].

A wide range of social, behavioral, and medical characteristics can potentially impact HRQoL. The same is true for African American older adults [2,11,12,13,14,15]. Low SES [16], health behaviors such as smoking [17], chronic disease [15], pain [18], and depression [2] all impact the HRQoL of African American adults. In one study, chronic medical conditions (CMCs) such as cancer, as well as symptom distress, depression, and functional status predicted the physical and mental HRQoL of African American older adults [2]. Although we know that multimorbidity and a higher number of CMCs reduce HRQoL, it is unknown which particular CMCs are independently linked to lower physical and mental HRQoL of African American older adults in economically disadvantaged urban areas.

Little information exists on the links between CMCs (other than cardiometabolic disease and cancer) and HRQoL among African American older adults in urban settings [19,20,21,22,23]. One of the areas that needs further attention is chronic respiratory conditions (CRCs). If CRCs are independent determinants of HRQoL among African American older adults (just as smoking is a risk factor for CRCs), then there is a need for greater investment in programs aimed at reducing smoking and CRCs in resource-scarce low-income urban African American communities. Given that smoking is a modifiable risk factor for CRCs [24,25,26,27], increased investment is needed to find the most effective smoking-cessation programs for African Americans [24,26]. Acceptable and expandable programs for reducing CRCs in such communities [23,28,29,30] are also needed.

Since smoking is associated with low HRQoL [31,32], a study investigating the relationship between CRCs and HRQoL must control for comorbid medical and mental conditions. Tobacco smoking is closely associated with CRCs, including asthma and chronic bronchitis [32,33]. Smoking also increases the risk of comorbid chronic medical and mental conditions [34] such as cancer [33,35,36], hypertension [37], heart disease [38,39], pneumonia [40,41], stroke [39], anxiety [42,43,44], depression [42], and mortality [45]. Similarly, such a study must also control for SES, a strong social determinant of health behaviors and HRQoL [46,47,48,49]. SES is linked to smoking [21,50], CRCs [51], and HRQoL [16]. Educational attainment and financial difficulty are additional social determinants that impact a wide range of health outcomes [46,52,53].

### 1.2. Aims

The current study tested the impact of CRCs on HRQoL in economically challenged African American older adults in South Los Angeles. To test this hypothesis, we controlled for potential confounders, such as demographic factors, SES, cigarette smoking, drinking, and comorbid medical and mental conditions that co-vary with CRC, as well as physical and mental HRQoL.

## 2. Methods

### 2.1. Design and Setting

A cross-sectional community-based survey was performed in South Los Angeles between 2015 and 2018 to investigate medication-related challenges of African American older adults with CMCs [54,55,56,57]. The survey included structured face-to-face interviews that collected extensive data on demographic factors (age and gender), SES (educational attainment and financial difficulty), living arrangements, marital status, health behaviors (cigarette smoking and alcohol consumption), health status (CRCs, comorbid CMCs, depressive symptoms, and pain intensity), and HRQoL (Physical and Mental Summary Scores; PCS and MCS). 

### 2.2. Participants and Sampling

Using a convenient sampling, a non-random sample (*n* = 617) of African American older adults was recruited from multiple predominantly African American housing units, senior centers, residential apartments, African American churches, and low-income housing projects located in Service Planning Areas (SPA) 6 of South Los Angeles. Participants were eligible if they were African American, non-institutionalized, and aged 65 years or older. Exclusion criteria included enrollment in skilled nursing facilities, enrollment in any other clinical trials, and considerable cognitive deficits. The populations of SPA1 to SPA8 are as follow: 334,951, 2,108,367, 1,846,997, 1,240,204, 646,770, 1,031,700, 1,369,589, and 1,596,245, respectively.

### 2.3. Institutional Review Board (IRB)

Charles R. Drew University of Medicine and Science (CDU IRB #: 14-12-2450-05) institutional review board (IRB) approved the study protocol. All participants signed a written informed consent before enrollment into this study. Participants received financial incentives for their participation.

### 2.4. Study Measures

*Demographic Characteristics.* Age and gender were the demographic covariates in this study. Age was treated as an interval variable. Gender was treated as a dichotomous variable.

*Socio-economic status (SES*). Two measures of SES were included in this study: educational attainment and financial difficulty. Educational attainment was measured as self-reported years of schooling and was operationalized as an interval variable, with a higher score indicating higher SES. Financial difficulty was measured using questions based on Pearlin’s list of chronic financial difficulties experienced by low SES individuals [58,59], such as not having enough money for essential needs like food, clothes, rent/mortgage, utility bills, etc. Using a 5-point Likert-type scale (“never” to “always”), a total financial difficulty score was calculated with a higher score reflecting lower SES (Cronbach’s alpha = 0.923).

*Living Arrangement and Family Type*. Two variables reflected each participant’s living arrangement and family type. A participant’s living arrangement was measured using a single item. Participants could state they were living alone or that they were living with others. Living arrangement and loneliness are strong determinants of health outcomes among older adults [60]. Family type was measured using a dichotomous variable, with “married” coded as 1 and “any other situation” coded as 0.

*Chronic Respiratory Conditions (CRCs).* We asked individuals if a physician had ever told them that they have asthma or chronic obstructive pulmonary disease.

*Comorbid Medical Conditions (CMCs).* Individuals were asked if a physician had ever told them that they have any of these conditions: heart disease, stroke, hypertension/high blood pressure, diabetes, cancer, back pain, arthritis, thyroid disorder, heart burn, and migraine. Self-reported assessment is a valid measure to collect data on CMC [61,62], however, some bias in estimates of this approach to measure multi-morbidity is expected. Our measure was reflective of the number of comorbid conditions.

*Depressive Symptoms.* This study used the 15-item Geriatric Depression Scale (Short Form) (GDS-SF) to evaluate depression [63]. Responses were on a yes/no scale. A sum score was calculated with a potential range between 0 and 15. A higher score was suggestive of the presence of more depressive symptoms. The GDS-SF has excellent reliability and validity. This measure has been extensively used to measure depression among older adults in both community and clinical settings [64,65].

*Pain Intensity.* We measured intensity of chronic pain by four subscales of the Short Form McGill Pain Questionnaire 2 (SF-MPQ-2) [66]. During a face-to-face interview, participants responded to 22 pain items that asked about the extent to which they experienced various types of pain in the past week. Each item was on an 11-point numeric rating scale from 0 (none) to 10 (worst possible). The subscales of the SF-MPQ-2 include (a) Continuity (throbbing, cramping, gnawing, aching, heavy, and tender pain), (b) Intermittence (shooting, stabbing, sharp, splitting, electric-shock, and piercing pain), (c) Neuropathic nature (hot-burning, cold-freezing, itching, tingling or “pins and needles,” light touch, and numbness pain), and (d) Affective domain (tiring-exhausting, sickening, fearful, and punishing-cruel pain). We calculated a total pain score, based on averaging responses to all questions [67,68]. A higher score is indicative of more intense chronic pain.

*Cigarette Smoking Status.* Participants were asked whether they smoke cigarettes using this single item question: “How would you describe your cigarette smoking habits?” Response items were “never smoked,” “previously smoked,” and “current smoker.” This variable was operationalized as a dichotomous variable (current smokers = 1, never/past smokers = 0).

*Drinking Status.* Participants were asked whether they drink alcohol. The exact question was “Do you drink alcohol?” Response items were yes and no. This variable was operationalized as a dichotomous variable (drinker 1, non-drinker 0).

*Physical and Mental HRQoL (PCS and MCS).* The HRQoL was measured using SF-12v2, which is a 12-item measure. This measure generates two summary scores and eight sub-domains (or subscales). Summary scores include the Physical Component Summary (PCS) and the Mental Component Summary (MCS) scores. Bodily Pain (BP), General Health (GH), Vitality (VT), and Social Functioning (SF) with one item each; and physical Functioning (PF), Mental Health (MH), Role Physical (RP), and Role Emotional (RE) domains, each with two items. To score the SF-12v2, we followed the method proposed by the original authors. The summary scores are calculated from z-scores of the 8 subscales. All scales contribute to the scorings of PCS and MCS, using weights from principal component analysis on the SF-36 scales. The norm-based scoring that is commonly used for SF-12v2 produces scores with a mean of 50 and a standard deviation of 10 for the US population. A higher score indicates better HRQoL [69,70,71,72,73,74].

### 2.5. Data Analysis

We used SPSS 23.0 (IBM Inc., Armonk, NY, USA) to conduct the data analysis. To describe the characteristics of our sample in the pooled sample and in those with and without a CRC, we used frequency (*n*), relative frequency (%), mean, and standard deviations (SD). We used the independent samples t-test and Chi Square to compare those with and without CRCs for our study variables. We also used the Pearson correlation test (zero order correlation) to test the bivariate associations between all study variables. We applied linear regression models with PCS and MCS of the HRQoL as outcomes, CRC as the independent variable, and demographic factors (age and gender), SES (educational attainment and financial difficulty), living arrangement, marital status, health behaviors (cigarette smoking and alcohol drinking), and health status (comorbid CMCs, depressive symptoms, and pain intensity) as confounders. We selected our confounders based on our conceptual model and the literature review. Using variables as Enter (rather than forward or backward), confounders stayed in the final model regardless of their significance. To run linear regression models, first we ruled out the collinearity between our independent and covariates. We did not find any evidence suggesting colinear covariates. We also checked for the normal distribution of the residuals in our regression model. That criterion was also met as we did not find any evidence suggesting deviation of the distribution to the error terms in our regression. We reported regression coefficients (b), Standard Error (SE), 95% Confidence Intervals (95% CI), and *p* values.

## 3. Results

### 3.1. Descriptive Statistics

Table 1 describes the study variables in the sample. All participants were 65 years or older, with 74.0 years old being the average. Participants were mostly females (65.2%), lived alone (60.0%) and were non-married (85.9%). In total, 47.0% reported cigarette smoking in their lifetime. From all participants, 24.3% had a CRC.

Table 1 also shows the differences in the study variables between those with and those without a CRC. Compared to those without a CRC, those with a CRC reported higher comorbid medical conditions, depressive symptoms, and pain. Compared to those without a CRC, those with a CRC reported lower physical and mental HRQoL (PCS and MCS) as well. (Table 1)

### 3.2. Bivariate Analysis

Table 2. shows the results of bivariate correlations between the study variables. This table reports Pearson correlation coefficients and *p*-value levels. Cigarette smoking and having or not having a CRC were positively correlated. MCS and PCS were not correlated. Having a CRC was negatively associated with MCS and PCS.

### 3.3. Multivariable Analysis

Table 3. shows the results of two linear regression models, separately for PCS and MCS as outcomes. These models show that having or not having a CRC is associated with lower PCS but not MCS, independent of confounders. Other factors that were associated with PCS included gender, financial difficulty, cigarette smoking, depressive symptoms, pain intensity, and number of medical comorbidities. Other factors that were associated with MCS included educational attainment, depressive symptoms, and pain intensity.

## 4. Discussion

In line with our hypothesis, we found that among African American older adults, having a CRC is associated with lower PCS (physical HRQoL) and MCS (mental HRQoL). The association between having a CRC and physical HRQoL (PCS) is independent of all confounders such as demographic factors, SES, cigarette smoking, presence of comorbid CMCs, pain, or depressive symptoms. The association between having a CRC and mental HRQoL (MCS) is due to confounders. 

Even after controlling for SES, cigarette smoking, depression, comorbid medical conditions (that include cardiometabolic disorders and cancer), and other possible confounders, having a CRC was found to be associated with lower physical HRQoL. This finding is in line with previous research in other race and ethnic groups [75]. This seemingly unique role of CRCs on HRQoL may be due to symptoms and disability that follow CRCs [76]. Individuals with CRCs suffer breathlessness, cough, and other respiratory symptoms, all of which reduce HRQoL [77]. Improvement of breathlessness is also shown to improve HRQoL [78]. Particularly in the presence of sudden attacks and exacerbations that can result in hospitalization and emergency department visits, some individuals with a CRC find their illness to be life threatening. All these physiological and psychological processes may explain why CRCs reduce PCS in African American older adults. However, neither CRCs nor comorbid medical conditions were independently associated with mental HRQoL in older African American adults. Why that is the case remains an unanswered question.

PCS was influenced by financial difficulty but not educational attainment. Multiple studies have shown that financial difficulty has considerable health implications for African Americans [79] and older adults [80]. In the African American community, financial difficulty predicts subjective and objective aspects of health, including chronic disease [81]. Financial difficulty is more detrimental to health when people do not have access to tangible social support, which can have a buffering effect [82]. This is probably why the PCS of African American older adults is more negatively influenced by financial difficulty.

Although in bivariate association, CRC was associated with worse MCS (mental HRQoL), this association did not remain significant in the multivariable analysis that controlled for our confounders. This suggests that confounders such as SES, cigarette smoking, or depression, or other factors may explain why African American older adults with a CRC report a worse mental HRQoL than those without a CRC. In other words, CRC is not an independent contributor to MCS, as it is to PCS.

We observed the effect of CRC on HRQoL after controlling for SES. In the general population [82], and among older adults [83], low SES increases the risk of conditions such as heart disease and cancer [84,85]. Financial difficulty operates as a stressor and increases oxidative stress [86]. It limits available choices that are needed to maintain health [83]. Low SES increases the risk of depression [79], cigarette smoking [87], alcohol use [88], CMC [81], and poor self-rated health (SRH) [89], which may confound the effect of CRC on HRQoL. The previous studies on cigarette smoking [87] and alcohol use [88] are among adults rather than African American older adults.

### 4.1. Implications for Health and Public Policy

The role of CRC as a cause of low HRQoL in African American older adults seems to be overlooked. This is in part because most of the attention in health disparities research is paid to cardiovascular disease, metabolic disease, and cancer in African Americans [90,91,92,93,94]. CRCs in African American children, youth, and young adults also receive some attention [95,96,97]. We argue that CRCs such as asthma and COPD in African Americans need more attention.

These results highlight a need for prevention of CRCs as a strategy for improving the HRQoL of older African American adults, particularly those who are of low SES and continue to smoke. Older African American adults often do not have access to smoking cessation programs. Our findings also suggest that policy makers should focus on prevention of CRCs, in addition to the current efforts aimed at reducing obesity, heart disease, diabetes, stroke, hypertension, and cancer. Without addressing CRCs, African American older adults may continue to have low HRQoL levels.

As smoking is a modifiable risk factor for CRC, there is a need to invest in reducing smoking in low-income African American communities, particularly those with limited resources. For several reasons, such as predatory and targeted advertisements, availability of tobacco outlets, differential patterns of initiation, as well as lower likelihood of access to cessation services, African Americans show an increased vulnerability to cigarette smoking [98,99,100,101,102,103,104,105,106,107,108,109,110,111,112,113,114,115,116,117,118]. African Americans who engage in substance use more rapidly transition to undesired trajectories and outcomes, a phenomenon called a telescoping effect. As a result, they are more strongly affected by the consequences of the substances they use [98,99,100,101,102,103,104,105,106,107,108,109,110,111,112,113,114,115,116,117,118]. For African Americans, the problem of cigarette smoking is beyond differences in prevalence but also extends to increased vulnerability to its effects [98,99,100,101,102,103,104,105,106,107,108,109,110,111,112,113,114,115,116,117,118].

### 4.2. Limitations

The current study is not without methodological limitations. First, due to a cross-sectional design, we cannot make causal inferences from the observed associations. Second, the sampling was not random. As a result, we cannot generalize our results to the entire population of African American older adults. In addition, we did not have data on individual income levels.

One major problem in this study was with the measurement of the independent variables, being CRC and comorbid CMCs. Our measures of CRC and CMC were self-reported and prone to measurement bias, both over-report and under-report. There is a need to validate these measures using administrative reports, claim data, or pharmacy data. We also did not have detailed data on the type and duration of CRCs. Finally, the study only included African Americans. The findings may differ for biracial or multi-racial African Americans. Also, our research did not consider the effect of air quality and air pollution on the observed relationships. More research is needed, including research with more detailed information about race, ethnicity and local environments. Despite these limitations, the results still contribute to the literature, as we do now know much more about the impact of having a CRC on the HRQoL of African American older adults.

### 4.3. Future Research

There is a need to study how density of pollutants and toxins and other risk factors (e.g., use of other substances) contribute to CRCs in African American older adults. As our sample was mainly low-income African Americans who lived in economically disadvantaged areas, there is a need to study multilevel determinants of CRCs, including closeness to traffic, air pollution, and second-hand smoke exposure. Future research may also explore how the risk of having a CRC increases when African Americans turn to cigarette smoking to cope with stress.

The results of the current study are specific to African American older adults in economically challenged urban areas, which might be very different from other economic, ethnic, socioeconomic, and geographic groups. These results may not apply to middle class or rural African Americans or those who are younger.

## 5. Conclusions

The presence of Chronic Respiratory Conditions (CRCs) seems to contribute to the poor physical HRQoL of African American older adults who live in economically disadvantaged urban areas. This effect is not entirely due to SES, cigarette smoking, and medical and mental comorbid conditions. There is a need to increase investment aimed at the treatment of CRCs and smoking cessation in the African American population. There is also a need to enhance disease management of African Americans with CRCs. Such strategies may have implications for the reduction of disparities in HRQoL in this population. More research is needed on the type, medications, and prognosis of CRCs such as asthma and chronic bronchitis.

## Figures and Tables

**Table 1:** Descriptive Statistics (*n* = 617).

Characteristics	All		CRC −		CRC +	
	*Mean*	*SD*	*Mean*	*SD*	*Mean*	*SD*
Age (Years) *	74.0	7.0	74.4	7.3	72.8	5.9
Educational Attainment (Years)	12.7	2.4	12.8	2.3	12.5	2.4
Financial Difficulty *	8.2	4.9	7.7	4.7	9.6	5.2
Comorbid Medical Conditions *	3.8	1.9	3.36	1.66	4.25	1.80
Depressive Symptoms *	2.1	2.4	1.8	2.2	3.0	2.9
Chronic Pain *	1.8	2.1	1.6	2.0	2.5	2.3
PCS HRQoL *	48.0	12.1	43.1	11.4	35.6	12.2
MCS HRQoL *	53.2	9.4	54.1	9.2	51.7	10.9
	*n*	*%*	*n*	*%*	*n*	*%*
Gender						
Women	402	65.2	309	66.2	93	62.0
Men	215	34.8	158	33.8	57	38.0
Family Type						
Non-Married	530	85.9	396	84.8	134	89.3
Married	87	14.1	71	15.2	16	10.7
Living Arrangement (Living Alone) ^#^						
No	247	40.0	197	42.2	50	33.3
Yes	370	60.0	270	57.8	100	66.7
Cigarette Smoking (Current) *						
No	327	53.0	278	59.5	49	32.7
Yes	290	47.0	189	40.5	101	67.3
Alcohol Drinking						
No	431	69.9	328	70.2	103	68.7
Yes	186	30.1	139	29.8	47	31.3
Asthma Bronchitis						
No	467	75.7	467	100.0	-	-
Yes	150	24.3	-	-	150	100.0

SD: Standard Deviation; CRC: Chronic Respiratory Conditions; HRQoL: Health-Related Quality of Life; PCS: Physical Component Summary Score; MCS: Mental Component Summary Score; ^#^ *p* < 0.1; * *p* < 0.05.

**Table 2:** Bivariate correlations.

Characteristics	1	2	3	4	5	6	7	8	9	10	11	12	13
1 Age (Years)	1	0.07	−0.19 **	−0.09 *	−0.05	−0.12 **	−0.13 **	−0.10 *	−0.12 **	0.04	−0.10 *	0.03	0.08 *
2 Gender ^a^		1	0.14 **	−0.02	−0.12 **	−0.20 **	−0.01	−0.02	0.08	0.12 **	−0.04	0.08	−0.16 **
3 Educational Attainment (Years)			1	−0.13 **	0.08	−0.09 *	0.07	−0.07	−0.03	−0.09 *	−0.05	0.15 **	−0.01
4 Financial Difficulty				1	−0.09 *	0.28 **	0.12 **	0.31 **	0.29 **	0.20 **	0.16 **	−0.24 **	−0.29 **
5 Married ^a^					1	−0.06	−0.08	−0.06	−0.08	−0.01	−0.06	0.00	0.11 **
6 Smoking Cigarette (Current) ^a^						1	0.21**	0.20 **	0.11 **	0.10 *	0.23 **	−0.12 **	−0.19 **
7 Drinking Alcohol ^a^							1	0.06	0.11 **	−0.02	−0.01	−0.03	−0.07
8 Depressive Symptoms								1	0.41 **	0.30 **	0.20 **	−0.51 **	−0.35 **
9 Pain Intensity									1	0.43 **	0.19 **	−0.30 **	−0.50 **
10 Comorbidities										1	0.22 **	−0.17 **	−0.45 **
11 CRC (Asthma, Chronic Bronchitis, or Emphysema)											1	−0.11 **	−0.27 **
12 MCS HRQoL												1	−0.04
13 PCS HRQoL													1

CRC: Chronic Respiratory Conditions; HRQoL: Health-Related Quality of Life; PCS Physical Component Summary Score; MCS: Mental Component Summary Score; * *p* < 0.05; ** *p* < 0.01; ^a^ Dichotomous Variables.

**Table 3:** Summary of two multivariable linear regression models with physical and mental HRQoL as outcomes.

Characteristics	PCS			MCS		
	b	95% CI	*p*	b	95% CI	*p*
Age	0.03	(−0.08, 0.14)	0.608	−0.03	(−0.13, 0.07)	0.600
Gender (Male)	3.07	(1.39, 4.76)	0.000	−1.17	(−2.62, 0.29)	0.116
Educational Attainment	−0.30	(−0.64, 0.05)	0.093	0.44	(0.14, 0.74)	0.004
Financial Difficulty	−0.22	(−0.39, −0.04)	0.014	−0.13	(−0.28, 0.01)	0.076
Married	1.89	(−0.35, 4.13)	0.099	−1.22	(−3.15, 0.72)	0.217
Cigarette Smoking	−2.30	(−3.99, −0.60)	0.008	0.38	(−1.08, 1.84)	0.611
Drinking	0.05	(−0.69, 0.79)	0.890	0.03	(−0.61, 0.67)	0.936
Depressive Symptoms	−0.40	(−0.76, −0.03)	0.033	−1.79	(−2.11, −1.48)	0.000
Pain Intensity	−1.72	(−2.16, −1.28)	0.000	−0.52	(−0.90, −0.14)	0.007
Comorbid Medical Conditions	−1.61	(−2.11, −1.10)	0.000	0.14	(−0.30, 0.57)	0.539
CRC (Asthma, Chronic Bronchitis, or Emphysema)	−3.00	(−4.89, −1.11)	0.002	0.21	(−1.42, 1.84)	0.800
Constant	54.63	(43.69, 65.57)	0.000	55.55	(46.11, 64.99)	0.000

CRC: Chronic Respiratory Conditions; HRQoL: Health-Related Quality of Life; PCS Physical Component Summary Score; MCS: Mental Component Summary Score.
